# Selective Oxidation of Glycolaldehyde to Glyoxal Using Ruthenium Complex Catalysts

**DOI:** 10.1002/cplu.202500616

**Published:** 2026-04-30

**Authors:** Takuya Sagawa, Atsushi Kondo, Mineo Hashizume

**Affiliations:** ^1^ Department of Industrial Chemistry Faculty of Engineering Tokyo University of Science Katsushika‐ku Japan; ^2^ Graduate School of Engineering Tokyo University of Science Katsushika‐ku Japan

**Keywords:** biomass, glycolaldehyde, glyoxal, oxidation, ruthenium complex catalyst

## Abstract

Cellulose comprises glucose units, and therefore glucose and its derivatives have received attention as a carbon source replacing fossil fuels. In particular, glyoxal, which is obtained from glucose by retro‐aldol reaction and subsequent oxidation via glycolaldehyde, is a feedstock for useful chemicals. However, efficient catalytic synthesis of glyoxal from glycolaldehyde has not been reported because the unwanted excessive oxidation of glyoxal occurs. In this study, catalytic synthesis of glyoxal from glycolaldehyde using a ruthenium complex catalyst with a bromophenyl terpyridine ligand was carried out. After optimizing reaction conditions, glyoxal was obtained in a 32% yield in *N*,*N*‐dimethylformamide at 100°C for 3 h using O_2_ gas as an oxidant. Furthermore, the obtained mixture was reacted with sodium sulfite to form precipitates, which are bisulfite adducts. It could be easily separated as a glyoxal equivalent from the catalyst and solvent by filtration. These results indicate that a new method for the synthesis of glyoxal from biomass‐derived glycolaldehyde has been achieved.

## Graphical abstract

Catalytic synthesis of glyoxal from glycolaldehyde using a ruthenium (II) complex catalyst and O_2_ gas was performed. Glyoxal yield reached 32%, and further oxidation of glyoxal was negligible. Furthermore, the obtained mixture containing glyoxal was precipitated by reaction with sodium sulfite, and it was confirmed that the sulfite adduct can be separated from the catalyst, solvent, and byproducts by filtration.

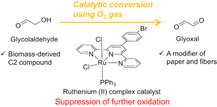

## 1 Introduction

Woody biomass is the most abundant on earth and is composed of cellulose, hemicellulose, and lignin [1, 2]. In particular, cellulose is one of the main components of the woody biomass and is a polymer of glucose constructed with 1,4‐β‐glycosidic bonds (Figure S1A, B). Therefore, cellulose can be used as a feedstock for organic chemicals, which is an alternative to fossil fuels [3, 4, 5, 6, 7]. Accordingly, the effective conversion of cellulose has attracted attention.

Glycosidic bonds of cellulose are cleaved by a Brønsted acid or base to form glucose. The production of glucose and cello oligosaccharides from cellulose by hydrolysis using mineral acids has been reported [8]. In recent years, heterogeneous catalysts have been used in the effective hydrolysis of cellulose [9, 10, 11]. Glucose has long been used as a raw material for various key chemical products [12, 13, 14, 15, 16, 17, 18, 19, 20, 21]. For example, ethanol is synthesized from glucose by an enzymatic method [12]. Recently, the catalytic conversion of glucose to adipic acid by dehydrogenation [13], hydroxymethyl furfural by trimolecular dehydration condensation [14, 15], sorbitol by hydrogenation [16, 17], and isosorbide by hydrogenation and subsequent dehydration condensation [18, 19] has been reported, all of which are used as raw materials for bioplastics. Furthermore, various compounds of C1 to C4 from glucose or its isomer have been synthesized by retro‐aldol reaction (Figure S1C) [20, 21, 22]. These products obtained by the industrial method are synthesized from fossil fuels, and therefore, the synthesis of C1 to C4 chemicals from glucose has received attention.

**FIGURE 1:**
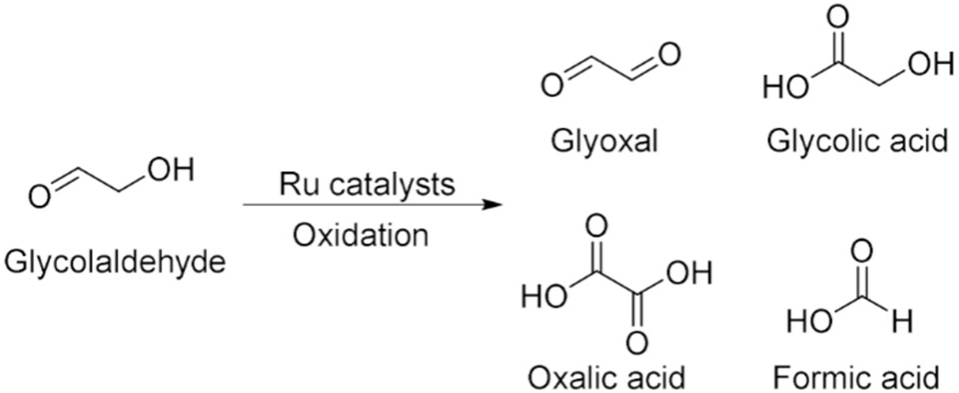
Oxidation of glycolaldehyde by Ru catalysts.

Among the products of retro‐aldol reaction from glucose, glycolaldehyde is a C2 compound having one hydroxy group and one formyl group and is a raw material for various compounds [23, 24, 25]. Reduction of the formyl group in glycolaldehyde to an alcohol gives ethylene glycol. It can be achieved by hydrogenation using a supported metal catalyst, and the reaction has been reported as a one‐pot reaction by a retro‐aldol reaction of glucose, followed by reduction [26, 27]. Furthermore, glyoxal, glycolic acid, and oxalic acid can be obtained by oxidation of glycolaldehyde [28, 29, 30]. In particular, glyoxal has been utilized as a modifier of papers and fibers. Recently, biobased wood adhesives, lignin‐glyoxal, have been developed for the replacement of conventional petroleum‐based wood adhesives comprising phenol and formaldehyde [31]. The stoichiometric synthesis of glyoxal from glycolaldehyde has been reported using glycerol oxidase or the PdCl_2_–CuCl_2_/[BMim]Cl system in 100% and 91% yield, respectively [32, 33]. However, efficient catalytic synthesis of glyoxal by catalytic oxidation from glycolaldehyde has not been achieved. To obtain glyoxal from glycolaldehyde, oxidation of the hydroxy group and suppression of further oxidation of formyl groups are necessary. A formyl group undergoes hydration in water to form geminal diols, which are easily oxidized to carboxylic acids. In particular, glyoxal is easily formed into the geminal diols, so that carboxylic acids such as glycolic acid, glyoxylic acid, and oxalic acid are easily generated (Figure 1). Thus, glycolaldehyde oxidation using oxidants such as H_2_O_2_ [30] and HNO_3_ [34] underwent excess oxidation to form corresponding carboxylic acids. Therefore, the development of efficient synthesis procedures for glyoxal *via* catalytic oxidation from glycolaldehyde is still desired.

To prevent further oxidation, a mild oxidation reaction in an organic solvent is required. Ruthenium‐oxo complexes are known as mild alcohol oxidation catalysts [35, 36, 37, 38, 39]. Fukuzumi, Kojima, and coworkers have reported the oxidation of various alcohols to corresponding aldehydes by ruthenium (IV)‐oxo complexes with pyridylamine as an ancillary ligand [35]. Further, Larihi and coworkers developed ruthenium (II) complex‐catalyzed oxidation of primary alcohols using oxygen gas [37]. In this study, catalytic synthesis of glyoxal from glycolaldehyde by divalent ruthenium complex catalyst with a 4′‐(4‐bromophenyl) 2,2′:6′, 2′’‐terpyridine (Br‐Ph‐tpy) ligand (RuCl_2_(PPh_3_)(Br‐Ph‐tpy), denoted as **1**) was conducted. The obtained glyoxal was reacted with Na_2_SO_3_ to form sodium sulfite ester as a precipitate, which was separated from the catalyst and solvent by filtration and evaluated by ^1^H NMR measurements.

## 2 Results and Discussion

### 2.1 Synthesis of Ruthenium Complex Catalyst 1

**FIGURE 2:**
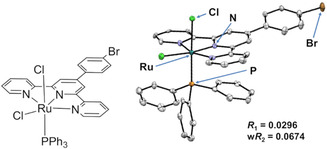
The structure of ruthenium complex catalyst **1** (left) and ORTEP drawing of **1** with thermal ellipsoids shown at 50% probably level. Hydrogen atoms are omitted for clarity (right).

A ruthenium complex catalyst **1** for the oxidation of glycolaldehyde was synthesized by mixing ruthenium complex, tris(triphenylphosphine)‐dichlororuthenium (II) (Ru(PPh_3_)_3_Cl_2_), and 4′‐(4‐bromophenyl)2,2′:6′, 2″‐terpyridine (Br‐Ph‐tpy) [40]. For effective oxidation of glycolaldehyde to form glyoxal, a precursor to the ruthenium–oxo complex was employed. It has been reported that the saturated ruthenium (II) complex [RuH(CO)Cl(PPh_3_)_3_] with PPh_3_ forms a ruthenium (IV)‐oxo complex via elimination of a PPh_3_ ligand with oxygen gas, which can oxidize various alcohols to aldehydes [37]. In addition, the use of a multidentate ligand as an ancillary ligand is expected to provide an improvement in the stability of the complex and no inhibition of the target reaction. Furthermore, it is also expected to provide further functionalities to the Br‐Ph‐tpy ligated complex as the bromophenyl group can serve as a substrate for Suzuki–Miyaura cross‐coupling and other reactions [41, 42]. For these reasons, the ruthenium (II) complex **1** with a PPh_3_ and Br‐Ph‐tpy ligand was chosen. The obtained complex **1** was characterized by NMR spectra and MALDI‐TOF‐MS (Figures S2, S3). Moreover, single crystal X‐ray structural analysis was carried out (Figure 2, Table S1). The crystal structure was an octahedral hexa‐coordination structure similar to a ruthenium complex coordinated 6,6‐bis(mesitylamino)‐terpyridine ligand, which does not contain a bromo group [43].

### 2.2 Confirmation of Oxidation Products of Glycolaldehyde

The products of glycolaldehyde oxidation were quantified by HPLC analysis. First, glycolaldehyde is converted to glyoxal or glycolic acid by oxidation. As further oxidation progresses, glyoxylic acid and oxalic acid are formed (Figure 1). Furthermore, oxalic acid is converted to formic acid and carbon dioxide. These products, excluding carbon dioxide, were separated by HPLC. The retention times of glycolaldehyde, glyoxal, glycolic acid, glyoxylic acid, oxalic acid, and formic acid were 14.1, 11.8, 14.8, 11.6, 8.8, and 16.4 min, respectively (Figure S4). Among them, the peaks of glyoxal and glyoxylic acid overlapped, and these were recognized by the UV detector (220 nm). Glyoxal does not absorb UV light at 220 nm, while glyoxylic acid exhibits an absorption peak at 220 nm derived from the π–π* transition of the carbonyl group (Figure S4A). Therefore, the inclusion of glyoxylic acid can be confirmed by the UV detector. Notably, no peaks derived from glyoxylic acid appeared under any reaction conditions. Thus, the peak appearing at 11.8 min (RI detector) is derived from glyoxal (Figure S4B), and the glyoxal yield was calculated from the peak. In addition, glyoxal was polymerized with a little water to form polyglyoxal [44]. Actually, it was confirmed that the obtained glyoxal was oligomerized by HPLC analysis, and the oligomer was depolymerized by heating the mixture. Furthermore, formic acid was confirmed as a product of glyoxal oxidation. It suggested that glyoxylic acid was produced as an intermediate; however, it could not be detected because of its immediate consumption. Moreover, carbon dioxide was confirmed using a gas detector (data not shown). Unidentified products were classified as “Others” (Table 1), and the generated carbon dioxide is included in “Others.“

**TABLE 1:** Oxidation of glycol aldehyde by ruthenium catalysts.

Entry^a^	Catalyst	Conversion/ %	Yield/ %					
			Glyoxal	Glycolic acid	Glyoxylic acid	Oxalic acid	Formic acid	Others
1	No cat.	49	3.8	2.8	<0.1	<0.1	6.8	36
2	RuCl_2_(PPh_3_)_3_	79	5.8	1.9	<0.1	<0.1	3.6	68
3	RuCl_3_•xH_2_O	74	12	3.0	<0.1	<0.1	5.3	54
4^b^	Ru/Al_2_O_3_	83	2.1	2.8	<0.1	<0.1	12	66
5	**1**	68	32	<0.1	<0.1	<0.1	0.9	35
6^c^	**1**	8.7	6.9	<0.1	<0.1	<0.1	<0.1	1.8
7^d^	**1**	62	12	11	<0.1	<0.1	13	26
8^d^^,^ e	**1**	60	4.0	9.0	<0.1	<0.1	23	24

a Conditions: glycolaldehyde 30 mg (0.50 mmol), catalyst 0.01 mmol (S/C = 50), solvent (DMF) 1.5 mL, oxidant (O_2_ gas) 0.25 MPa, time 3 h, temperature 100°C.

b Ru/Al_2_O_3_ 0.6 mg (S/C = 50).

c Solvent (H_2_O) 1.5 mL.

d Solvent (acetonitrile) 1.5 mL.

e Oxidant (TBHP aq.) 1.0 mmol.

### 2.3 Screening of Ruthenium Catalysts

Screening of ruthenium catalysts for the synthesis of glyoxal through the oxidation reaction of glycolaldehyde was conducted (Figure 1). Oxygen gas was used as an oxidant, and the reaction was carried out in *N*,*N*‐dimethylformamide (DMF) at 100°C for 3 h. When no catalyst was used, 49% of glycolaldehyde was consumed, and 3.8% of oxides of glycolaldehyde, such as glyoxal, were obtained (Table 1, Entry 1). It is suggested that this is due to auto‐oxidation caused by oxygen [45]. In the case of using ruthenium trichloride or Ru(PPh_3_)_3_Cl_2_, the conversion of glycolaldehyde and yield of the products were almost the same as without a catalyst (Entry 2, 3). These conditions provided formic acid with 3.6% (Entry 2) and 5.3% (Entry 3), while glyoxylic acid and oxalic acid were not obtained. Therefore, glyoxylic acid and oxalic acid might be consumed immediately in these conditions. When Ru/Al_2_O_3_ was used, a few glyoxal and further oxidized compounds were obtained (Entry 4). The formyl group of glyoxal was hydrated easily to form geminal diol, which undergoes further oxidation to give carboxylic acids. On the other hand, the ruthenium complex catalyst **1** provided 32% yield of glyoxal (turn over number (TON) is **≈**16), and the yield of further oxidized compounds was negligible. Further, glyoxylic acid was not formed when using **1** as a catalyst. The desired compound, glyoxal, can be obtained by oxidation of the hydroxy group in glycolaldehyde. It suggested that the ruthenium (IV)‐oxo complex was formed from **1**, and the complex oxidized the hydroxy group in glycolaldehyde to form glyoxal. The details of the proposed reaction mechanism were explained later. From the present results, glyoxal can be obtained with the highest yield when the ruthenium complex catalyst **1** was used.

### 2.4 Effects of Solvent and Oxidant

The effects of solvents and oxidants on the oxidation reaction of glycolaldehyde regarding catalyst **1** were confirmed. Water, acetonitrile, and DMF were used as solvents. When water was used, a small amount of glyoxal was produced (6.9%), and further oxides were not obtained due to the poor solubility of catalyst **1** in water (Table 1, Entry 6). On the other hand, a large amount of carboxylic acid was produced when acetonitrile was used (Entry 7). Meanwhile, a 30% yield of glyoxal was obtained when DMF was used (Entry 5). Catalyst **1** is activated by the elimination of the PPh_3_ ligand and subsequent formation of the ruthenium–oxo complex. As a result, the oxidation of the hydroxy group proceeded. However, acetonitrile can coordinate with the ruthenium complex catalyst, resulting in a suppression of the coordination of hydroxy groups. Therefore, oxidation of glycolaldehyde to glyoxal in acetonitrile did not proceed sufficiently. Furthermore, the reason why carboxylic acids such as glycolic acid and formic acid were produced in acetonitrile was due to the high solubility of oxygen gas. It has been reported that the O_2_ gas dissolved at an oxygen partial pressure of 0.212 bar is 77.4 mg/L in acetonitrile at 25°C, which is higher than that in DMF (33.7 mg/L) [46, 47]. As a result, the glycolaldehyde oxidation in acetonitrile provided corresponding carboxylic acids. Moreover, the investigation of the effects of oxidants on the oxidation of glycolaldehyde was also conducted. When using TBHP aq., a few amount of glyoxal was obtained, and further oxidized compounds such as glycolic acid were formed by water in TBHP (Entry 8). Accordingly, we chose oxygen gas as the oxidizing agent and DMF as the solvent.

### 2.5 Time Course and Byproducts

**FIGURE 3:**
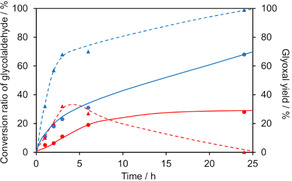
Time course of glycolaldehyde oxidation at 70°C (circles and solid lines) and 100°C (triangles and dotted lines). Blue: conversion ratio of glycolaldehyde. Red: glyoxal yield.

To investigate the influence of reaction temperature and reaction time on the oxidation of glycolaldehyde, the time courses of the glycolaldehyde conversion and glyoxal yield with different temperatures were confirmed (Figure 3). When the reaction was carried out at 70°C, the conversion ratio gradually increased with reaction time, and the glyoxal yield reached 28% after 24 h. On the other hand, the reaction temperature at 100°C showed a significant improvement of the glycolaldehyde conversion by 32% after an hour and 68% after 3 h. However, the increase in reaction time decreased the glyoxal yield. The yield was 32% after 3 h; however, it was 27% after 6 h and less than 0.1% after 24 h. It suggested that longer reaction times lead to further oxidation, and in particular, formic acid was obtained with a yield of 15% for 24 h. Consequently, increasing the reaction temperature improved the conversion ratio and glyoxal yield; however, prolonged reaction time caused a decrease in glyoxal yield due to excessive oxidation.

To clarify the byproducts in the oxidation of glycolaldehyde, oxidation reactions using glyoxal and formic acid as substrates were performed. When the reaction temperature was 70°C, the conversion of glyoxal was 7.8%, and small amounts of formic acid were formed (Table S2, Entry 1). Meanwhile, the reaction temperature at 100°C showed consumption of glyoxal at 81% (Table S2, Entry 2), indicating that higher temperatures produced further oxidation of glyoxal. To confirm other products of these reactions, formic acid was further oxidized. Formic acid was consumed with 54% and 99% at 70°C and 100°C (Table S2, Entry 3,4), respectively, and carbon dioxide was produced in both cases detected by a gas detection tube (data not shown).

### 2.6 Separation of Glyoxal

**FIGURE 4:**
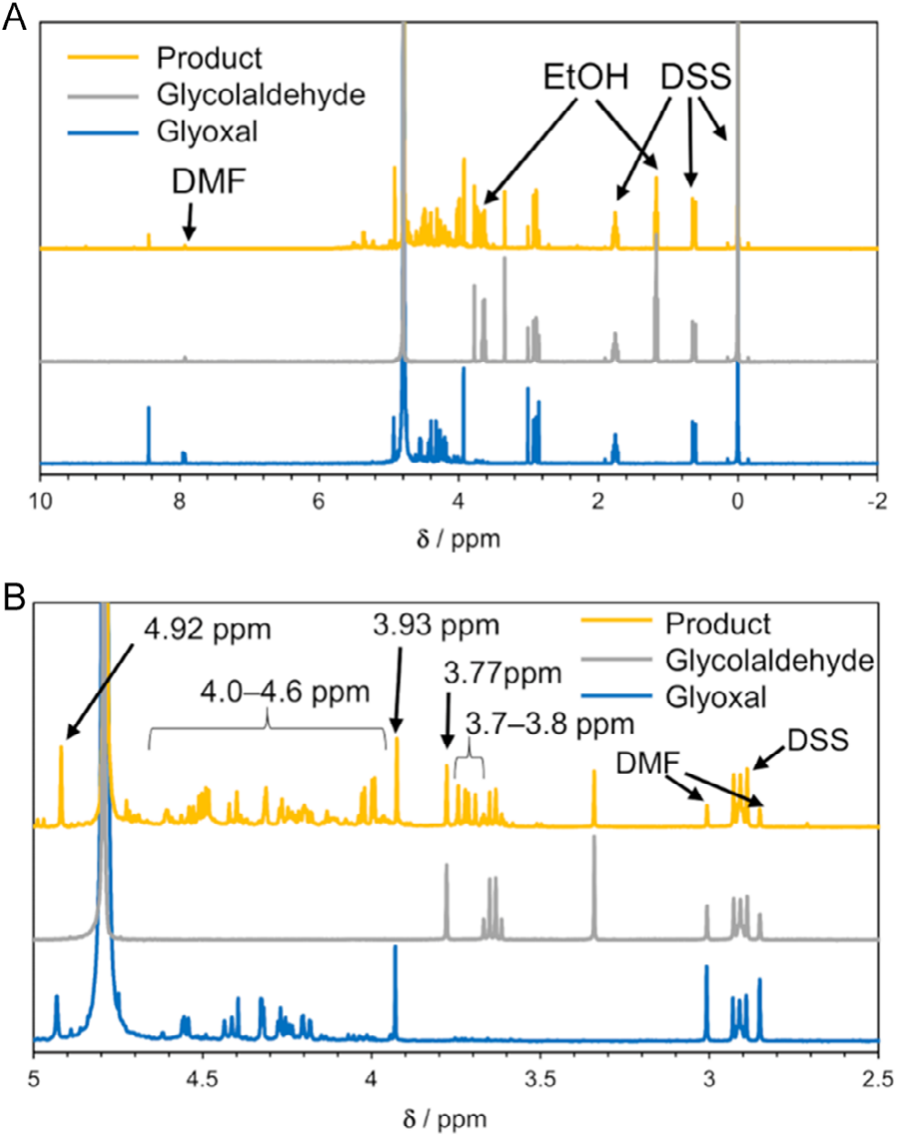
^1^H NMR spectra of sodium bisulfate adduct of the mixture of glycolaldehyde oxidation, glycolaldehyde, and glyoxal. (A) overall view and (B) enlarged view.

In conventional methods, glyoxal has been synthesized by the oxidation of ethylene glycol and purified by distillation. On the other hand, the purification of glyoxal from the reaction mixture, which is the product of the oxidation of glycolaldehyde, is generally difficult because of the generation of various undesired byproducts like glycolic acid and formic acid. Here, the glyoxal bisulfite adduct was obtained using sodium sulfite for simple separation, similar to fatty bisulfite adducts [48]. When aldehyde substrates are mixed with sodium hydrogen sulfite or sodium sulfite, the bisulfite adducts are formed [48, 49, 50]. The bisulfite adducts are insoluble in organic solvents such as DMF, and therefore the obtained glyoxal can be separated easily by filtration from organic solvents, catalysts, and byproducts. The formation of the bisulfite adduct was carried out under the conditions with the highest glyoxal yield (Table 1, Entry 5), and a white solid was obtained. In entry 5, 32% of glyoxal and 0.9% of formic acid were formed, and 32% of glycolaldehyde remained. Therefore, the white solid should include reaction products, which are glyoxal sodium bisulfite and glycolaldehyde sodium sulfite (Figure S5). The white solid was analysed by ^1^H NMR spectrum measurements (Figure 4), and signals originating from glyoxal sodium bisulfite were observed at 4.92 ppm. Further, signals at 3.93 ppm, around 3.7–3.8 ppm, and around 4.0–4.6 ppm were characterized as glyoxal sodium bisulfite (oligomer). Moreover, a signal at 3.77 ppm appeared derived from glycolaldehyde sodium sulfite. Notably, signals originating from the ruthenium complex catalyst **1** did not appear. Based on these results, the obtained glyoxal sodium bisulfite can be separated from the reaction mixture that contained DMF, the catalyst **1,** and byproducts simply by filtration. Therefore, further separation and purification of glyoxal should become much easier by deprotection of bisulfite groups of the glyoxal derivatives with NaOH aq. and subsequent distillation.

### 2.7 Reaction Mechanisms

**FIGURE 5:**
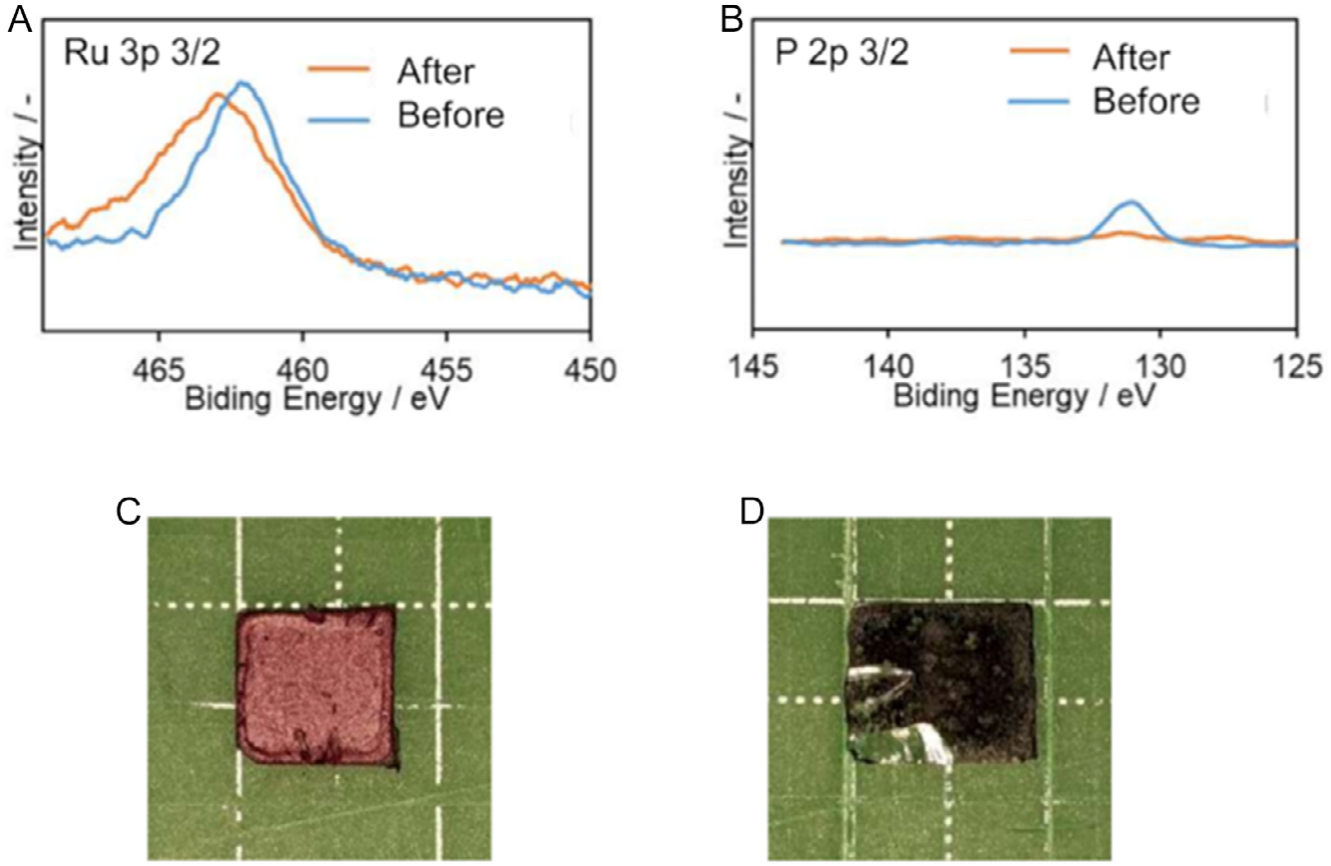
XPS spectra of **1** (A) Ru 3p 3/2 (B) P 2p 3/2. Photographs of ruthenium complex‐coated glass substrate (C) before oxidation and (D) after oxidation.

**FIGURE 6:**
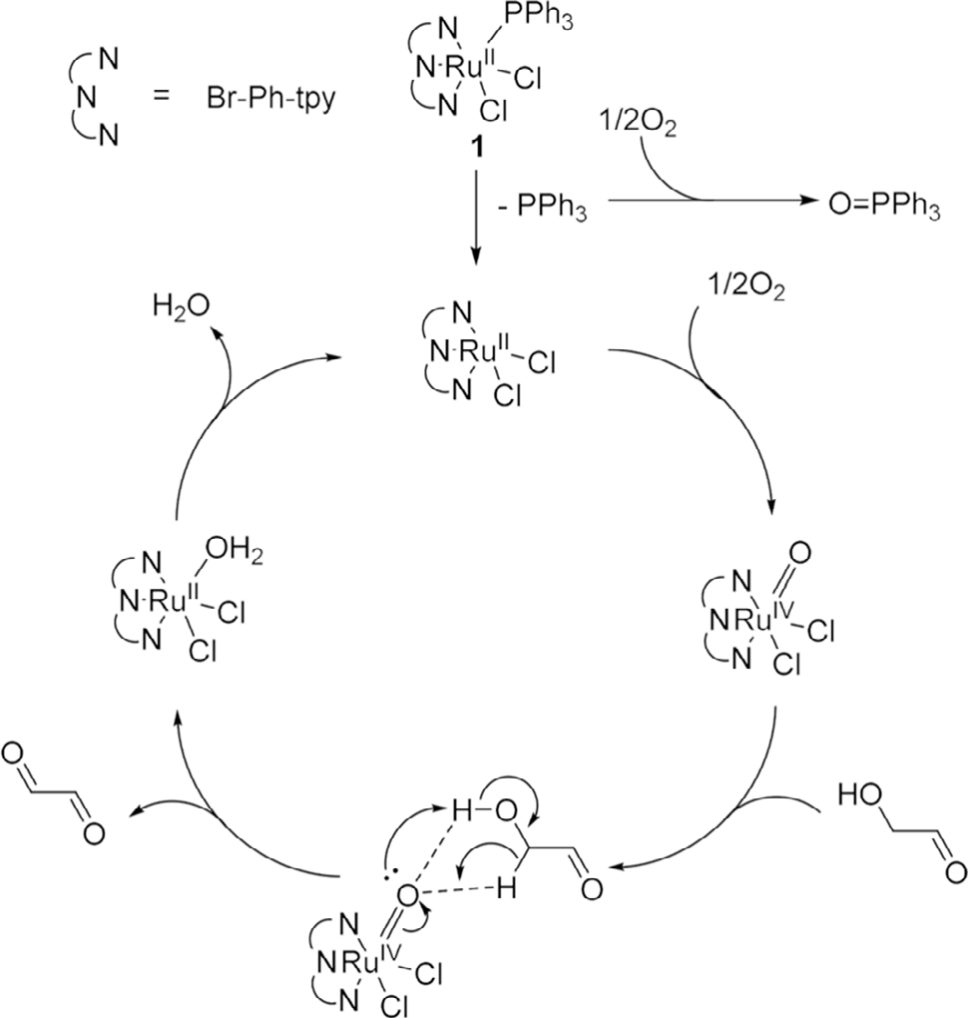
Proposed mechanism for oxidation of glycolaldehyde.

To elucidate the reaction mechanism of glyoxal oxidation by Ru complex catalyst **1**, XPS measurements of **1** before and after the oxidation reaction of glycolaldehyde were performed. The respective reaction mixture before and after oxidation was coated on glass substrates (Figure 5, S6), and the XPS spectra of Ru 3p 3/2, O 1s, Cl 2p 3/2, C 1s, N 1s, and P 2p 3/2 were measured. In the XPS spectra before oxidation, a peak originating from Ru 3p 3/2 appeared at 462.3 eV, while the peak after oxidation shifted to higher energy (462.8 eV, Figure 5A). Peaks originating from Ru (II) 3p 3/2 appeared between 461.2–462.6 eV, which are lower than that of Ru (IV) 3p 3/2 (463.2 eV) [51]. It indicated that the valence of the ruthenium center in **1** increased to four valences by oxidation to form a ruthenium–oxo complex. Further, the peak intensity originating from O 1s after oxidation was larger than before the reaction, suggesting that the oxygen atom derived from oxygen gas was ligated to the ruthenium center (Figure S6(A)). When a ruthenium–oxo complex is formed, the ligand in the complex before oxidation should be eliminated. Peaks originating from Cl 2p 3/2 and N 1s after oxidation were comparable to those before oxidation (Figure S6 (B), (C)). Meanwhile, a peak of P 2p 3/2 disappeared after oxidation (Figure 5B), indicating that **1** was activated by the elimination of PPh_3_, and the ruthenium–oxo complex was formed. Furthermore, it was confirmed that PPh_3_ was not coordinated to the ruthenium center after the ruthenium–oxo complex was formed. Moreover, the color of 1 after reaction was changed to dark gray from brown, which is consistent with the XPS results (Figure 5C,D). Based on the results and similar reports [36, 52], the reaction mechanism of glycolaldehyde by ruthenium complex catalyst 1 was proposed (Figure 6). First, the ruthenium–oxo complex (IV) was formed by elimination of the PPh_3_ ligand, and subsequent O atom was coordinated to the ruthenium (II) center. After that, glycolaldehyde was inserted, and a transition state with a five‐membered ring was generated. Then, glyoxal was generated and eliminated from the ruthenium complex catalyst, and the water molecule was coordinated to the ruthenium (II) center. Finally, the water molecule was eliminated by the reaction of O_2_ gas, and the ruthenium–oxo catalyst (IV) was regenerated. The mechanism is consistent with the XPS results.

## 3 Conclusion

Synthesis of glyoxal from glycolaldehyde using ruthenium complex catalyst **1** was demonstrated. Optimizing reaction conditions, glyoxal yield reached 32% with O_2_ gas as oxidant in DMF at 100°C for 3 h. **1** was reacted with O_2_ gas, and a ruthenium–oxo complex was generated, and the hydroxy group in glycolaldehyde was oxidized to the formyl group. Further, it was confirmed that the obtained mixture containing glyoxal was precipitated by reaction with sodium sulfite. The sulfite adduct can be separated from the catalyst, solvent, and byproducts by filtration. These results indicate a new method for the synthesis of glyoxal from glycolaldehyde, which can be obtained from glucose derived from woody biomass. The new synthesis method can replace the current method using fossil fuels and is expected to reduce environmental impact.

## 4 Experimental Section

### 4.1 Materials

Oxalic acid, formic acid (98%), RuCl_3_·nH_2_O, triphenylphosphine (PPh_3_), glycolic acid, ruthenium alumina (Ru 5%, Ru/Al_2_O_3_), and Na_2_SO_3_ were purchased from FUJIFILM Wako Pure Chemical Corporation. Glyoxal (39% aqueous solution), glyoxylic acid (50% aqueous solution), *tert*‐butyl hydroperoxide (70% in water, TBHP aq.), and 4′‐(4‐bromophenyl)2,2′:6′, 2″‐terpyridine (Br‐Ph‐tpy) were purchased from Tokyo Chemical Industry. DMF and diethyl ether (Et_2_O) were purchased from Nacarai Tesque Co. Ltd. Glycolaldehyde and acetonitrile were purchased from Sigma–Aldrich. Distilled water and ultrapure water (18.2 MΩ•cm) prepared by Advantec RFD210TA and Advantec RFD414BA, respectively, were used in the experiments.

### 4.2 Preparation of Tris(triphenylphosphine)‐Dichlororuthenium (II) (Ru(PPh_3_)_3_Cl_2_)

Ru(PPh_3_)_3_Cl_2_ was prepared by the previous procedure with several modifications [53]. RuCl_3_·nH_2_O (100 mg, 0.38mmol; calculated as RuCl_3_·3H_2_O) and PPh_3_ (600 mg, 2.29 mmol) were dissolved in methanol, and the mixture was refluxed for 3 h. After cooling down to room temperature, the mixture was filtered and washed with methanol and Et_2_O. The obtained products were dried under reduced pressure, and brown powder (Ru(PPh_3_)_3_Cl_2_; 320 mg, 86%) was obtained.

^31^P NMR (162 MHz, CDCl_3_): δ 28.99.

### 4.3 Preparation of the Ruthenium Complex Catalyst **1**

The synthesis was conducted by a previous method with several modifications [40]. Ru(PPh_3_)_3_Cl_2_ (147 mg, 0.15 mmol) and Br‐Ph‐tpy (59 mg, 0.15 mmol) were added to toluene (15 mL) and refluxed for 18 h. The crude product was washed with toluene and Et_2_O and collected by filtration. The obtained product was dried under reduced pressure, and the desired Ru complex was obtained as a purple solid (112 mg, 90%). The obtained Ru complex **1** was characterized by NMR and MALDI‐TOF‐MS (Figures S2 and S3).

^1^H NMR (400 MHz, CDCl_3_): δ 7.00 (td, *J* = 2.0, 7.5 Hz, 6H, CH), 7.10 (td, *J* = 1.4, 7.4 Hz, 3H, CH), 7.20–7.30 (m), 7.31 (m, *J* = 6.6 Hz, 2H, CH), 7.44 (d, *J* = 8.7 Hz, 2H, CH), 7.48 (d, *J* = 8.5 Hz, 2H, CH), 7,55 (s, 2H, CH), 7.58 (m, *J* = 8.3 Hz, 2H, CH), 7.73 (d, *J* = 8.1 Hz, 2H, CH), 9.44 (d, *J* = 5.2 Hz, 2H, CH). ^31^P NMR (162 MHz, CDCl_3_): δ 40.7. MALDI‐TOF‐MS calcd. for C_39_H_30_BrClN_3_PRu 786.00 [M−Cl]^+^; found 785.50.

Crystal data: Monoclinic; space group *P* 2_1_; *a* = 11.181 (2) Å, *b* = 12.274(2) Å, *c* = 30.900 (6) Å; *V* = 4223.5 (13) Å^3^; *Z* = 4; *ρ*_calcd_ = 1.526 g cm^−^^3^; μ = 0.118 mm^−^^1^; 2*θ*_max_ = 53.42°; reflections collected: 45 312, independent reflections: 14 833 (*R*_int_ = 0.0337), *R*_1_(*I* > 2σ) = 0.0296, *wR*_2_(*I* > 2σ) = 0.0674. Deposition Number https://www.ccdc.cam.ac.uk/services/structures?id=doi:10.1002/cplu.202500616R1 2 293 499 (for **1**) contains the supplementary crystallographic data for this paper. These data can be obtained free of charge from The Cambridge Crystallographic Data Centre and Fachinformationszentrum Karlsruhe http://www.ccdc.cam.ac.uk/structures Access Structures service [54].

### 4.4 Oxidation of Glycolaldehyde by Ru Catalysts

Glycolaldehyde (0.50 mmol) and the catalyst (RuCl_2_(PPh_3_)_3_, RuCl_3_•xH_2_O, Ru/Al_2_O_3_, and **1**) were dissolved in 1.5 mL of solvent in a portable reactor (TVS‐N2, Taiatsu Glass Kogyo, Co. Ltd., 10 mL), and O_2_ gas was pressurized to 0.25 MPa. Then, the apparatus was put into an oil bath to keep the reaction temperature at 100°C. After cooling down the reactor, N_2_ gas was bubbled for 30 s to substitute the O_2_ gas. Water (0.5 mL) was added to the mixture to analyse soluble products by a high‐performance liquid chromatograph (HPLC) equipped with HPX‐87H column (Aminex, mobile phase: 0.5 mM H_2_SO_4_ aq. 0.5 mL min^−1^, 35°C). Yields of glyoxal, glycolic acid, glyoxylic acid, oxalic acid, and formic acid were determined by an absolute calibration method using the purchased compounds as standards. Furthermore, oxidation without catalysts and with TBHP aq. instead of O_2_ gas was also carried out as a control experiment. Moreover, a separation of the obtained glyoxal by sodium sulfite was conducted as follows. Saturated sodium sulfite aqueous solution (0.1 mL) was added to the obtained mixture to generate white precipitation. Afterward, ethanol (2.0 mL) was added and decanted to obtain the white solid. The white solid was washed with ethanol and dried under reduced pressure. The white solid was characterized by ^1^H NMR spectrum.

### 4.5 Oxidation of Glyoxal and Formic Acid

Oxidation reactions of glyoxal and formic acid were performed in a similar manner to those of glycolaldehyde. Glyoxal (0.50 mmol) and formic acid (0.50 mmol) were used instead of glycolaldehyde (0.50 mmol), and the reaction was performed at 70°C or 100°C for 3 h. After the reaction, the mixture was analysed by HPLC equipped with HPX‐87H column. The gas phase was analysed by a gas detector (GV‐110S, GASTEC) with a gas‐detecting tube for CO_2_ (2LC, GASTEC).

### 4.6 Characterization

NMR spectra were recorded on an AVANCE 400 M spectrometer (Bruker). ^1^H NMR (400 MHz) and ^31^P NMR (162 MHz) spectra were measured using 4,4‐dimethyl‐4‐silapentane‐1‐sulphonic acid sodium salt (DSS) as an external standard, and 85% phosphoric acid was used as an external standard for ^31^P NMR (162 MHz). Matrix‐assisted laser desorption/ionization time‐of‐flight mass spectrometry (MALDI‐TOF‐MS) was recorded on a JMS‐S3000 (JEOL). X‐ray photoelectron spectroscopy (XPS) was recorded on a JPS‐9030 (JEOL). A single crystal of the Ru complex was prepared by recrystallization from its DMSO/CHCl_3_ solution. Crystal data were collected on a D8 QUEST (Bruker) with graphite‐monochromated Mo Kα radiation (*λ* = 0.71073 Å) at 90 K. The crystal structure was solved by the intrinsic phasing method (SHELXT‐2014) [55] and refined by full‐matrix least‐squares methods on F2 (SHELXL‐2016) [56] with APEX 4 software. The nonhydrogen atoms were refined anisotropically, and the hydrogen atoms were refined isotropically. The obtained structure is shown in Figure 2.

## Supporting Information

Additional supporting information can be found online in the Supporting Information section.

## Funding

This study was supported by Japan Society for the Promotion of Science (19K15608, 23K13780).

## Conflicts of Interest

The authors declare no conflicts of interest.

## Supporting information

Supplementary Material

## Data Availability

The data that support the findings of this study are available on request from the corresponding author. The data are not publicly available due to privacy or ethical restrictions.
