# Supplementary material for: Selective Oxidation of Glycolaldehyde to Glyoxal Using Ruthenium Complex Catalysts
Source: Chempluschem. 2026 Apr 30;91(5):e202500616. doi: 10.1002/cplu.202500616 (PMC13130154; doi:10.1002/cplu.202500616)
Supplement: Supplementary file 1 — Supplementary Material [file CPLU-91-e202500616-s001.pdf]

Catalytic Synthesis of Glyoxal by Oxidation of Glycolaldehyde Using Ruthenium Complex Catalysts

Takuya Sagawa,[1,2*] *Atsushi Kondo,*[2] *Mineo Hashizume*[1,2*]
[1]Department of Industrial Chemistry, Faculty of Engineering, Tokyo University of Science, 6-3-1 Niijuku, Katsushika-ku, Tokyo 125-8585, Japan.

[2]Graduate School of Engineering, Tokyo University of Science, 6-3-1 Niijuku, Katsushika-ku, Tokyo, 125-8585, Japan.

–Totals– 9 pages, 6 Figures, 2 Tables

Table of Contents

Figure S1 Epimerization of glucose and their cleavage to form C2 to C4 compounds by retro-aldol reaction. ············ S2
Figure S2 NMR spectra of ruthenium complex catalyst **1**. ············ S3
Figure S3 MALDI-TOF-MS of ruthenium complex catalyst **1**. ············ S4
Figure S4 HPLC chart of substrate and products of glycolaldehyde oxidation. ············ S5
Figure S5 Reaction scheme of glycolaldehyde or glyoxal with sodium bisulfite. ············ S6
Figure S6 XPS spectra of ruthenium complex catalyst **1** ············ S7
Table S1 Crystallographic data and structure refinement detail for ruthenium complex catalyst **1**. ············ S8
Table S2 Oxidation reactions using glyoxal and formic acid as a substrate. ············ S9

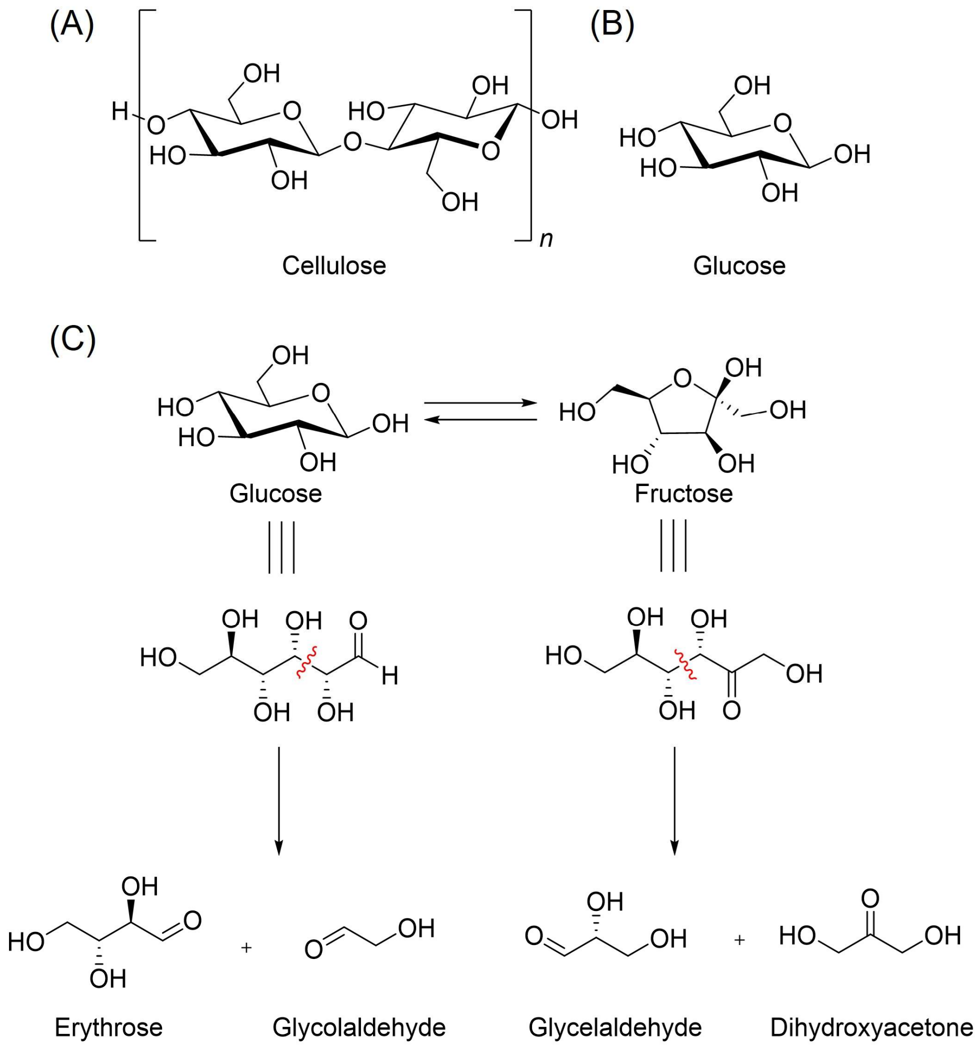

Figure S1. Structure of (A) cellulose and (B) glucose. (C) Epimerization of glucose and their cleavage to form C2 to C4 compounds by retro-aldol reaction.

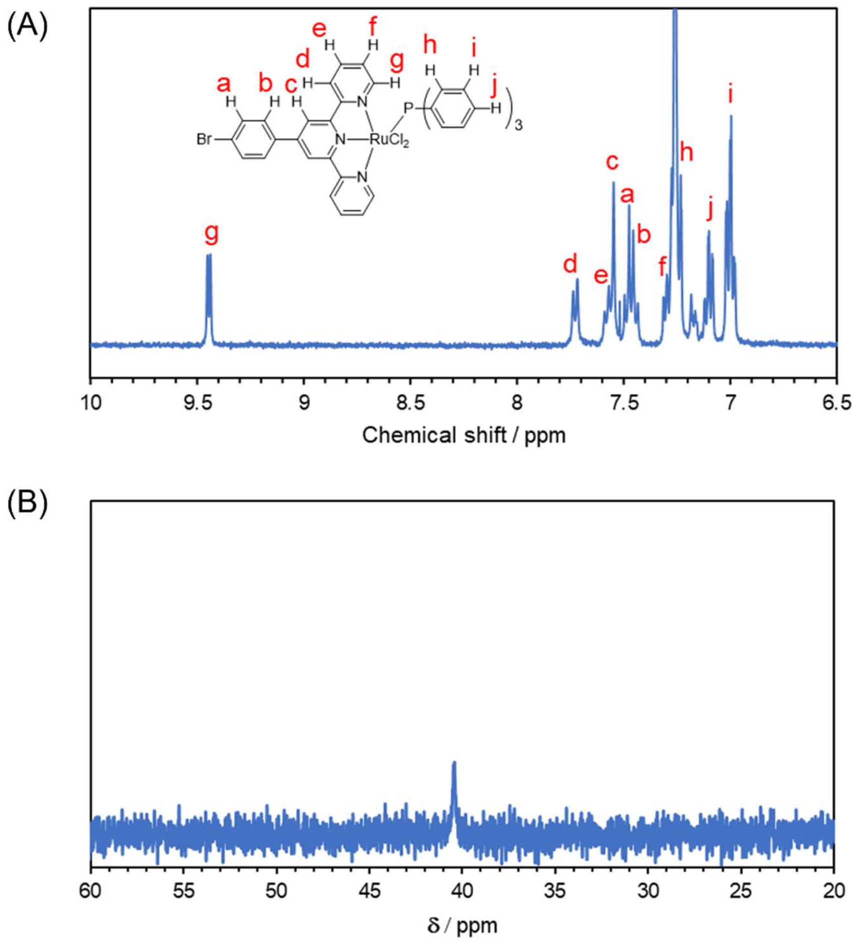

Figure S2. (A) ^{1}H NMR spectrum (6.5–10.0 ppm) and (B) ^{31}P NMR spectrum (20–60 ppm) of ruthenium complex catalyst **1**.

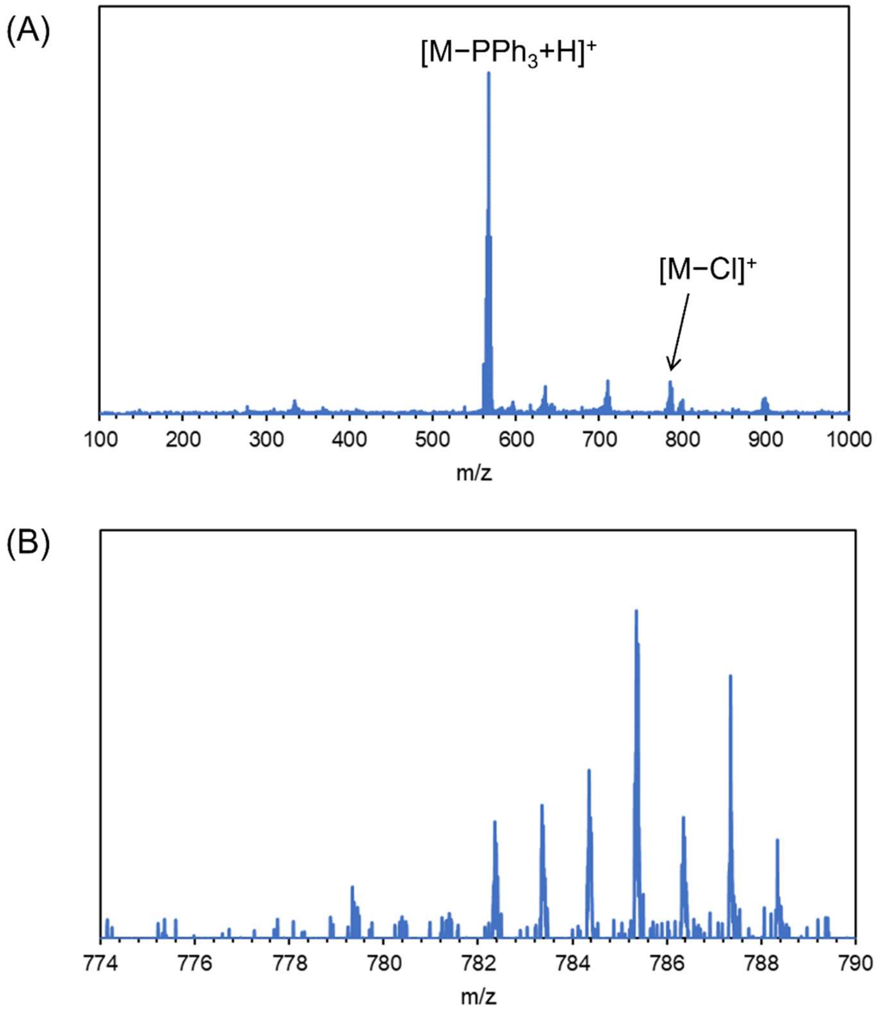

Figure S3. MALDI-TOF-MS of ruthenium complex catalyst **1**. (A) Overall view, (B) Enlarged view.

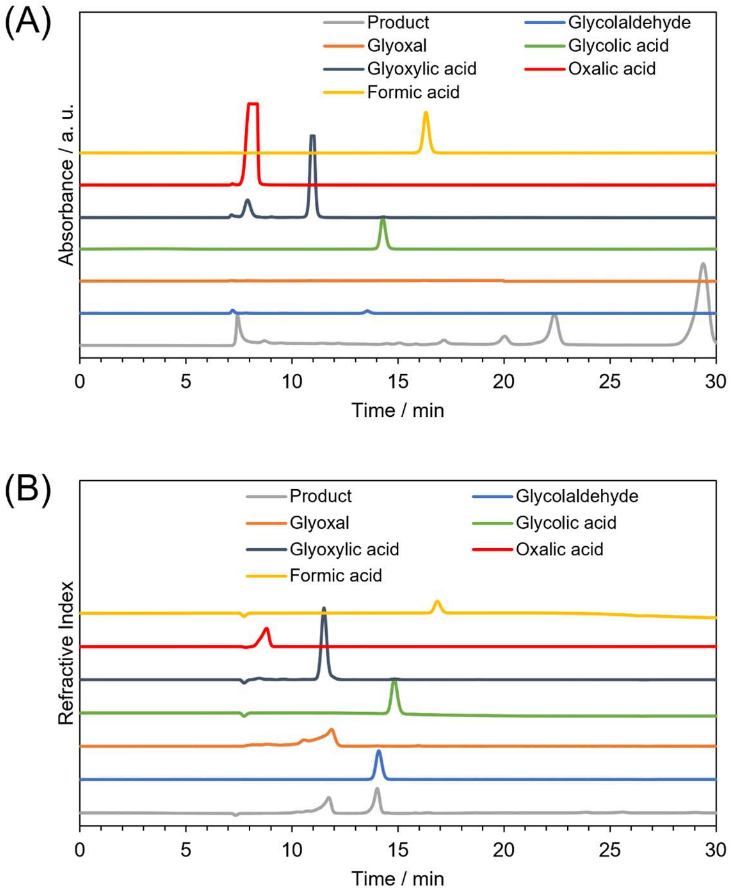

Figure S4. HPLC chart of substrate and products of glycolaldehyde oxidation using (A) UV detector, and (B) RI detector. Glycolaldehyde, glyoxal, glycolic acid, glyoxylic acid, oxalic acid, and formic acid were purchased compounds. “Product” was the obtained mixture of glycolaldehyde oxidation using ruthenium complex catalyst **1** (Table 1, Entry 5).

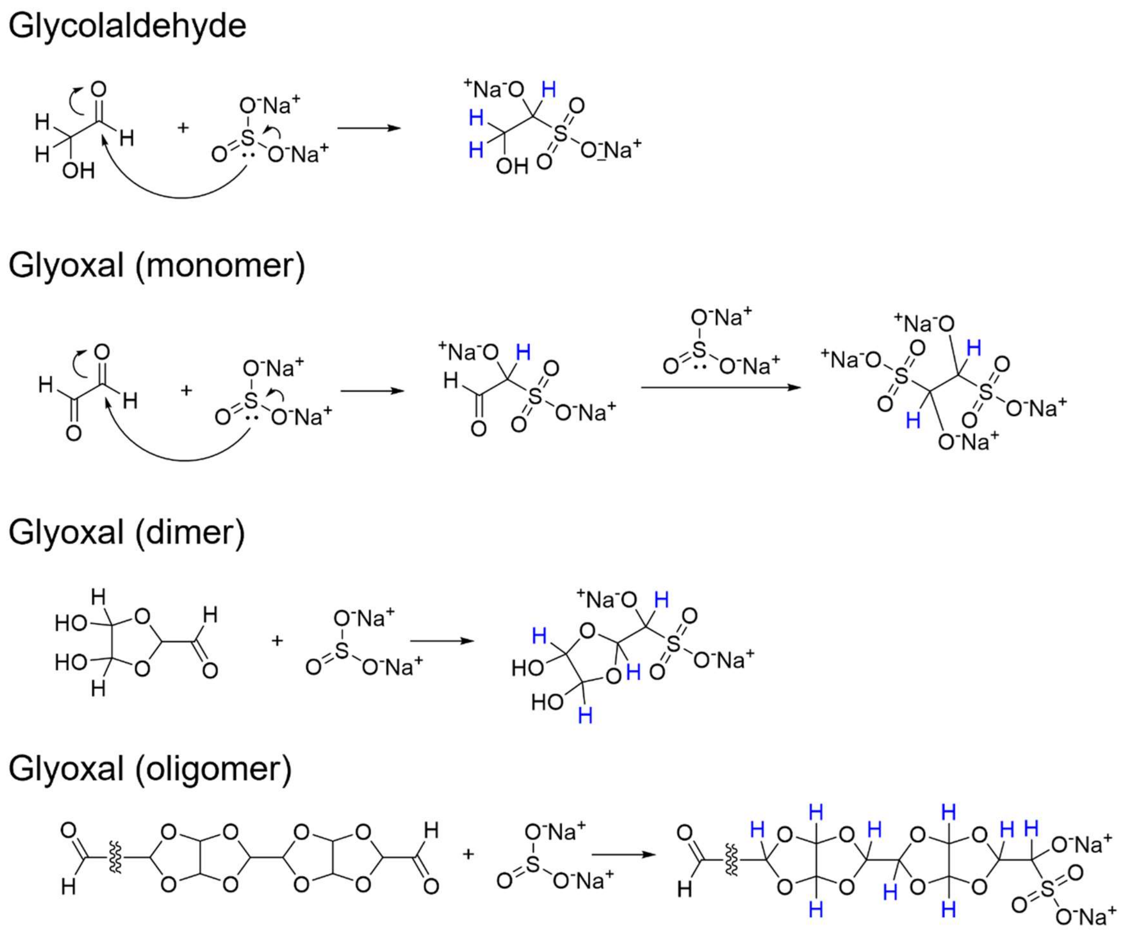

Figure S5. Reaction scheme of glycolaldehyde or glyoxal with sodium bisulfite.

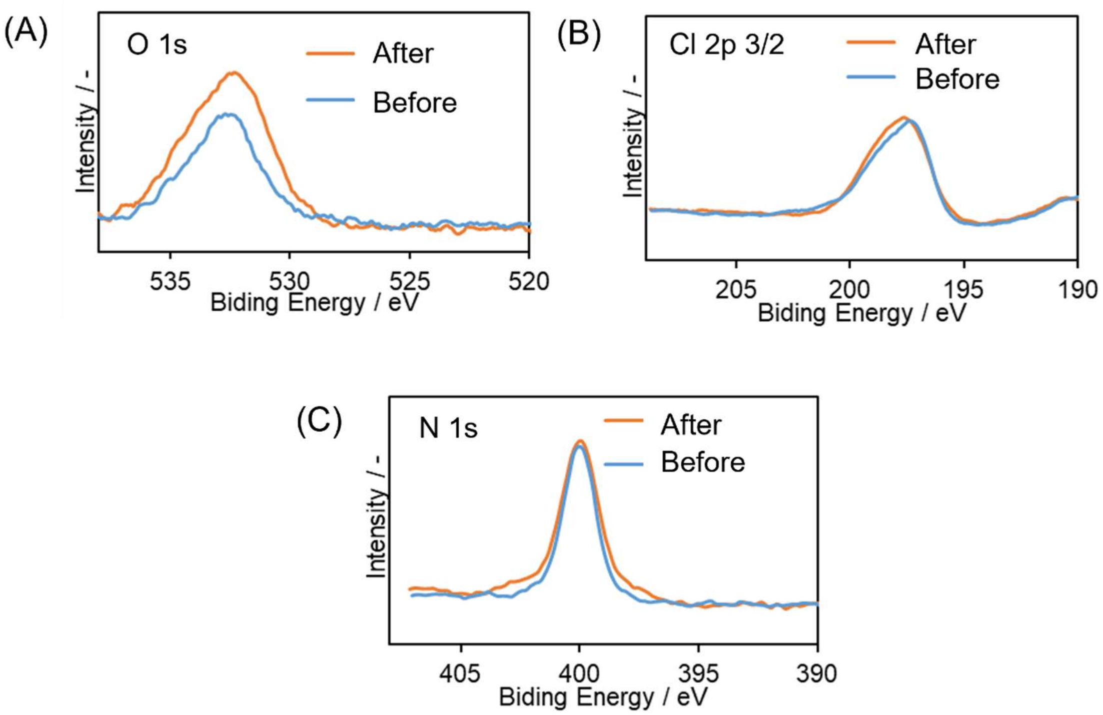

Figure S6. XPS spectra of ruthenium complex catalyst **1**. (A) O 1s, (B) Cl 2p3/2, (C) N 1s, and (D) P 2p3/2 regions.

Table S1. Crystallographic data and structure refinement detail for ruthenium complex catalyst **1**.

Emprical formula	$C_{45}H_{43}BrCl_2N_5O_2PRu$
Formula weight	968.68
T (K)	103
Wavekength (Å)	0.71073
Crystal system	monoclinic
Space group	P 1 21 1
a (Å)	11.181(2)
b (Å)	12.274(2)
c (Å)	30.900(6)
α (deg)	90
β (deg)	84.85(3)
ν (deg)	90
V (Å^3)	4223.5(13)
Z	4
D_{calc} (g/m^3)	1.526
F (000)	1968
Reflection collected	45312
Indipendent reflections (R_{int})	14833 (0.0403)
R_1 ($I > 2\sigma(I)$)	0.0296
wR_2 (all data)	0.0674
Goodness of fit (GoF) on F^2	1.041

Table S2. Oxidation reactions using glyoxal and formic acid as a substrate.[a]

Entry	Substrate	Temperature/ °C	Conversion/ %	Yield/ %			
				Glyoxylic acid	Oxalic acid	Formic acid	Others
1	Glyoxal	70	7.8	< 0.1	< 0.1	1.4	6.4
2	Glyoxal	100	81	< 0.1	< 0.1	6.7	74
3	Formic acid	70	54	-	-	-	54
4	Formic acid	100	99	-	-	-	99

[a]Conditions: Substrate 0.50 mmol, Catalyst 0.01 mmol (S/C = 50), DMF 1.5 mL, O_2 gas 0.25 MPa, 3 h.
